# Enhanced Iteroparity Is a Correlated Response to Direct Selection on Blood Feeding in a Mosquito

**DOI:** 10.1002/ece3.71335

**Published:** 2025-04-28

**Authors:** Rudyard J. Borowczak, Mary A. Wood, William E. Bradshaw, Peter A. Armbruster, Christina M. Holzapfel

**Affiliations:** ^1^ Institute of Ecology and Evolution University of Oregon Eugene Oregon USA; ^2^ Department of Biology Georgetown University Washington DC USA

**Keywords:** bet‐hedging, genetic correlation, genotype‐by‐environment interaction, life‐history evolution, *Wyeomyia smithii*

## Abstract

Herein, we determine life‐history consequences of selection on blood feeding in a polymorphic population of the pitcher‐plant mosquito, *Wyeomyia smithii* Coq. (Diptera: Culicidae). All populations of *W. smithii* produce an initial batch of eggs without ever taking a blood meal (biting); southern populations require a blood meal for the second and subsequent batches of eggs, but are polymorphic for propensity to bite. To determine correlated life‐history responses to direct selection on blood feeding, we compared fecundity, adult longevity, and reproductive allocation between a line selected specifically for increased blood feeding and its unselected, control line maintained in parallel for 11 generations. Previous studies have focused on the fitness benefits of blood feeding in terms of overall fecundity. Herein, we evaluate a novel fitness benefit of blood feeding that reduces the risk of reproductive failure by spreading that risk across multiple reproductive events in a population confronted with an unpredictably variable larval environment. We propose that “spreading the risk” reinforces selection on blood feeding in other arthropods in which the separation of fecundity from reproductive allocation in time or space has previously been neglected. Importantly, heritable variation for “spreading the risk” should enhance vectorial capacity and make more difficult vector control through larval source reduction.

## Graphical abstract

Coq. (Diptera: Culicidae). Previous studies have focused on the fitness benefits of variable larval environment.

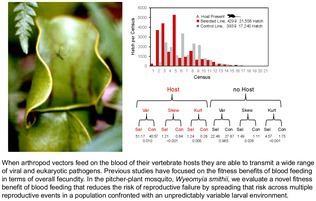

## 1 Introduction

Life histories are an accounting of the major components of fitness through time. The life histories of organisms represent a compromise or tradeoff among the maximization of survivorship and fecundity and the minimization of time. These tradeoffs are usually interpreted as adaptations to the historical and ecological context in which the organisms have evolved and currently live (Stearns 1976). Life‐history theories are unifying concepts of how fitness traits should vary individually or covary as functionally coordinated groups or “syndromes” (Dingle 1986; Buoro and Carlson 2014) along a continuum between extremes. Verification of these unifying concepts is, by the very nature of the continuum, comparative. Life‐history theory predicts how selection should shape the evolution of fitness traits and, therefore, should be tested by genetic differences in fitness traits along a selection gradient, in contrasting ecological contexts, or among selected lines. Herein, we utilize a southern population of the pitcher‐plant mosquito, *Wyeomyia smithii* (Coq.), to compare survivorship and reproduction through time, between a line selected specifically for avid biting and an unselected, reluctant‐biting line maintained in parallel without blood feeding (Bradshaw et al. 2018).

Iteroparous reproduction occurs when organisms reproduce in a pattern that is to some degree spread out in time or space.^1^ The contrasting pattern is semelparity, when organisms reproduce only once. But what degree of instantaneous reproduction represents true semelparity (Fritz et al. 1982; Kirkendall and Stenseth 1985)? We envision iteroparity as a continuous, quantitative trait, rather than as a discrete, antipolar comparison with semelparity. The essence of the matter is the distribution of reproductive effort in time and space over an adult lifespan (Fritz et al. 1982; Bradshaw 1986). Genotypes, populations, or species are then more or less iteroparous, not either iteroparous or semelparous.

If iteroparity represents the pattern of reproductive allocation, then the selective process leading to that pattern is included in Bonner's fundamental concept of “range variation,” where “the range of variation is genetically determined” (Bonner 1965, 95). In the real world, “local groups either die out, are started, or reach too high a number in an irregular spatial sequence which shifts again in time” (Den Boer 1968, 172). As a consequence, organisms are expected to a greater or lesser extent to “spread the risk” (Den Boer 1968) or “hedge their bets” against reproductive failure (Slatkin 1974; Stearns 1976; Fritz et al. 1982; Bradshaw 1986; Philippi and Seger 1989) by dispersing reproduction through time or space. Inherent in spreading the risk (bet‐hedging) is delaying the production or allocation of some offspring until more than one suitable habitat is encountered. Spreading the risk involves some degree of repeated reproductive events, that is, results in some degree of iteroparity. The connection between spreading the risk and iteroparity becomes apparent if each is envisioned as a continuous, quantitative trait. Herein, we show how genetic variation and covariation (Box 1) of blood feeding, adult female longevity, and iteroparity combine into a bet‐hedging syndrome in the pitcher‐pant mosquito, *W. smithii* , when viewed in the context of selection in the ecological background of a north Florida wet pine savannah.

Mosquitoes are able to transmit a wide variety of viral and eukaryotic pathogens when they bite and imbibe the blood of vertebrate hosts. However, three genera (*Toxorhynchites, Malaya, Topomyia*) never bite (Downes 1958; Foster 1995; Rattanarithikul et al. 2007; Wahid et al. 2007; Miyagi et al. 2012; Zhou et al. 2014), and the ability to mature eggs without biting has evolved independently multiple times in genera comprised mostly of species that are obligate biters (Spielman 1971; Rioux et al. 1975; O'Meara 1985a, 1985b). By contrast, there is only one species of mosquito that bites in one part of its range and is obligate non‐biting in the rest of its range: the pitcher‐plant mosquito, *W. smithii* (Smith and Brust 1971; Bradshaw 1980, 1986; O'Meara et al. 1981; Bradshaw and Holzapfel 1983a; Bradshaw et al. 2018, 2022). All populations of *W. smithii* are fully interfertile regardless of geographic origin or propensity to bite. Northern, obligate non‐biting populations are derived from more southern, biting ancestors (Bradshaw and Lounibos 1977; Armbruster et al. 1997, 1998, 1999; Mathias et al. 2006; Merz et al. 2013). In this paper, we are concerned with the life‐history consequences of selection on increased blood feeding (hereafter, biting) in a north‐Florida (southern) population of *W. smithii* that is polymorphic for propensity to bite.

*W. smithii* lives only in the water‐filled leaves of the carnivorous purple pitcher‐plant, *Sarracenia purpurea* ; their distribution extends from the Gulf and Atlantic Coasts of North America northwards to Newfoundland and westwards in Canada to Saskatchewan (Merz et al. 2013). The limiting resources for larval development are prey captured by individual leaves, which are maximally attractive to prey soon after the leaves open; thereafter, prey capture declines exponentially (Bradshaw 1983). In a Florida Gulf Coast population (30° N), competition among developing larvae is intense throughout all four seasons and results in only 12% of first‐instars ultimately pupating in nature (Bradshaw and Holzapfel 1986). Even among pupating *W. smithii* , increased larval density results in longer pre‐adult development, lower pupal weights, lower lifetime fecundity, and consequently, lower fitness (Istock et al. 1975; Moeur and Istock 1980; Bradshaw and Holzapfel 1989, 1990, 1992; Broberg and Bradshaw 1995). Hence, increased larval densities extract a cost to fitness not only directly through larval death, but also indirectly through unrealized reproductive potential (Bradshaw and Holzapfel 1992).

For organisms that complete their pre‐adult development in discrete habitats, intense resource competition places a premium on hatching into the larval habitat before other individuals—the importance of “being the first there”^2^ (Zwölfer 1979; McLachlan and Cantrell 1980; Livdahl 1982; Bradshaw 1986; Maciá and Bradshaw 2000). Among southern populations of *W. smithii* , blood‐feeding increases the degree of iteroparity (a phenotypic correlation), enabling adults to allocate reproduction to multiple habitats in time as well as space (Bradshaw 1986).

Box 1 **Correlated response to selection (Falconer 1981, eq. 19.6).**

1
$${\mathrm{CR}}_{Y}=\frac{S_{X}}{\delta_{\mathrm{PX}}}\left(h_{X}h_{Y}r_{A}\delta_{\mathrm{PY}}\right).$$

CR_Y_ = correlated response in trait Y to direct selection on trait X (herein, X = blood feeding; Y = adult longevity or iteroparity).

*S*_X_ = selection differential imposed on trait X.

*δ*_PX_ = phenotypic standard deviation in trait X.

*δ*_PY_ = phenotypic standard deviation in trait Y.

*h*_X_ = (heritability of trait X)^1/2^.

*h*_Y_ = (heritability of trait Y)^1/2^.

*r*_A_ = additive genetic correlation between trait X and trait Y.

“Estimates of genetic correlations are usually subject to large sampling errors and are therefore seldom very precise” (Falconer 1981, 184). Consequently, one is usually less interested in the exact numerical estimate of heritability or a genetic correlation, but rather whether the estimated heritabilities and genetic correlations are significantly nonzero, and also the sign of the genetic correlation. Such information can be derived from the correlated response of one trait to direct selection on a second trait. For there to be a correlated response, each of the elements in the parentheses must be non‐zero; consequently, if direct selection on blood feeding elicits a correlated response in a life‐history trait, then both of the heritabilities are significantly nonzero and there must be a significant non‐zero genetic correlation between them, with the sign of the genetic correlation being given by the sign of the correlated response.

**FIGURE 1:**
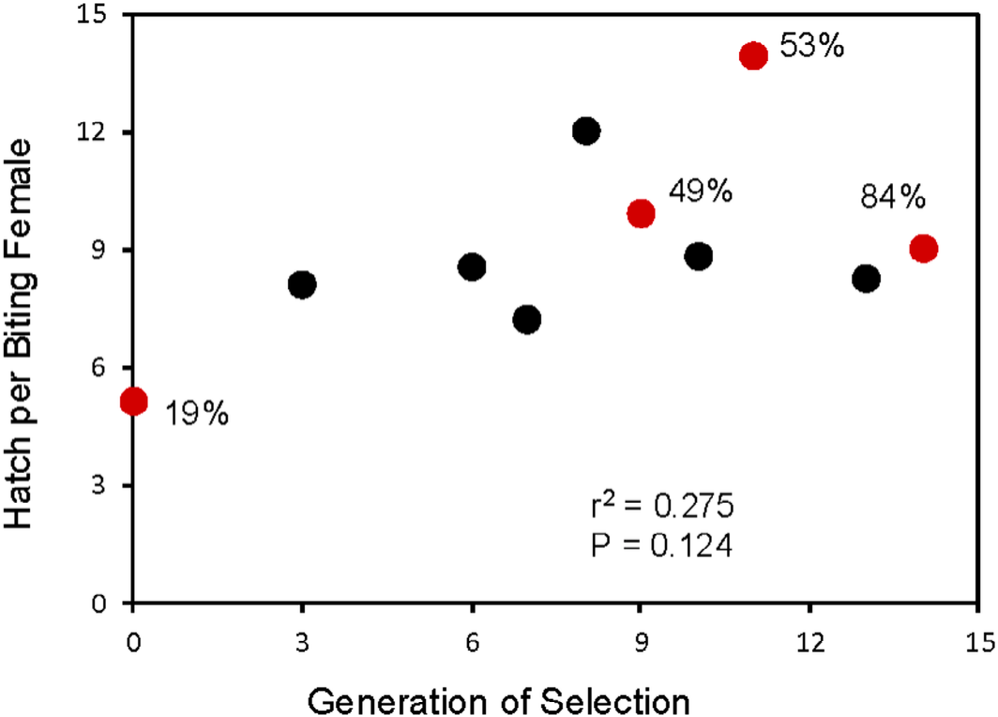
Hatch per biting female is not significantly correlated with direct selection on blood feeding (biting) in *Wyeomyia smithii* . Percentage of eclosed females that bit is given next to each red symbol. Compiled from Bradshaw et al. (2022, tables 2 & and s2).

Southern populations produce an initial batch of eggs without a blood meal but require a blood meal to mature the second and subsequent ovarian cycles (Bradshaw 1980, 1986; Moeur and Istock 1980; O'Meara et al. 1981; Mahmood and Crans 1999). The incidence of blood‐feeding may vary among southern populations (30°–35° N and < 300 m altitude); but, southern populations invariably produce their first and largest batch of eggs without biting (Bradshaw and Lounibos 1977; Bradshaw 1980; O'Meara and Lounibos 1981; O'Meara et al. 1981), precluding selection on non‐biting. Among a variety of mosquitoes, blood feeding individuals tend to achieve greater reproductive success than individuals from the same population that take a smaller number of blood meals or mature a batch of eggs without biting (Corbet 1967; Spielman 1971; O'Meara 1985a; Briegel and Hörler 1993). If blood feeding in *W. smithii* is a mechanism underlying increasing fecundity, fecundity should increase with generations of selection. In fact, after 14 generations of direct selection on blood feeding, the incidence of biting increases from an initial 21% to 84%, but per‐capita fecundity^3^ from blood feeding is only tenuously correlated with the incidence of blood feeding in the selected line (Figure 1). There is more in the game afoot; additional life‐history benefits of blood feeding reside elsewhere in *W. smithii* 's life cycle.

Herein, we propose that blood feeding enhances iteroparity within a population (phenotypic pattern) as a result of selection for spreading the risk of reproductive failure (evolutionary process) in an ecological context that varies in space and time. In a north Florida population (Wilma, 30° N), larval competition for limiting resources is intense. Throughout an entire growing season at Wilma, only 8%–18% of hatching first instars in 538 leaves actually pupated (Bradshaw and Holzapfel 1986). The first larvae to hatch in a leaf have the competitive advantage both of ‘being the first there’ (McLachlan and Cantrell 1980) and of the greatest expectation of future resources, since prey capture (the nutrient base of the community within pitcher‐plant leaves) is transitory, occurring predominantly in the youngest leaves and declining exponentially thereafter (Bradshaw and Holzapfel 1983a). Pitcher plants at Wilma are scattered about the wet pine savannah and average 0.08 freshly opened leaves per plant per day (Bradshaw and Holzapfel 1986). Hence, ovipositing females are presented with a mosaic of opportunities that vary in time as well as space (Bradshaw and Holzapfel 1983a).

If blood feeding is maintained as a correlated response to selection on a bet‐hedging strategy (Bradshaw 1986), direct selection for avid biting should result in a more prolonged reproductive life, thereby facilitating a greater dispersal of offspring than in an unselected control line with a low incidence of biting (genetic correlations). Simply put, if females do not live long enough, they can notspread the risk of reproductive failure in a mosaic of variable opportunities for oviposition. We test the first proposition by comparing the spread of reproduction within each of two lines: a line selected for avid biting and its unselected control line. We test the second proposition by comparing the spread of reproduction and adult female longevity between the selected and the control line.

### 1.1 Experimental Rationale

Previously, our laboratory has attempted to develop an avid biting line with a starting population of 1000 on three occasions. All failed due to an inability to impose repeated selection on a modest population with an initial low incidence of biting. The solution occurred when we were able to collect 14,000 individuals from a Florida population (30° N, locality Wilma). Consequently, we split this population into two lines: 5000 to be maintained as an unselected control line, 9000 to be selected for avid blood feeding (biting). Replicate selected and control lines would be ideal, but our goal was to develop an avid blood‐feeding line from within a population with a low incidence of biting (21%, Bradshaw et al. 2022). Hence, we focused our efforts on developing one line that could sustain itself by blood‐feeding alone. This focused approach was successful by the seventh generation of selection. Thereafter, we were able to use the excess for experiments, including the present determination of the life‐history consequences of selection on biting in a population polymorphic for that trait. It is important to note that this paper is based on the comparison between two lines: one line selected for avid biting, the other control line that sustained its original low incidence of biting (Bradshaw et al. 2022). Having previously used these lines to address questions about the role of drift vs. selection in the evolution of obligate non‐biting in nature (Bradshaw et al. 2018), here we take advantage of having finally developed an avid biting line to determine the life‐history consequences of a blood‐feeding lifestyle. We note that the large population sizes of both lines likely mitigate the contribution of drift to our results but acknowledge the need to interpret our novel results within the context of a comparison between a single control and selected line.

### 1.2 Variance as a Measure of Iteroparity

The underlying essence of theory relating to iteroparity or bet‐hedging reduces to the pattern of offspring production over time. Dispersion in the timing of reproduction has been measured by Kirkendall and Stenseth's (1985) index of iteroparity *θ*_*i*_ = *ω*/*α*, where *α* and *ω* are the ages of first and last reproduction, respectively. *θ*_*i*_ depends upon only two offspring, the first and the last, and ignores the number and timing of those in between. Bradshaw (1986) used a more inclusive index of iteroparity, Ip = (Ʃ*xp*_*x*_)/*α*, where *p*_*x*_ is the proportion of total propagules produced by individuals at age *x*. Ip is still scaled to the timing of the first offspring. Consequently, we measured the variation in the timing of reproduction simply as the variance in time from parent to offspring hatch summed over all hatches.

### 1.3 Specific Hypotheses at Risk

1. The variance in reproductive timing is greater in the selected line than in the control line, both in the presence and in the absence of a host.
2. If the first hypothesis is true, and there is a significant departure from an underlying normal distribution of reproductive timing in the selected line, then that departure will be reflected by increased positive skew or increased positive kurtosis. Positive skew results from a an extended tail to the right (later adult age); positive kurtosis results from a symmetrical repulsed or spread distribution relative to a normal distribution (Sokal and Rohlf 1995, 114). Either positive skew or positive kurtosis in the selected line would reflect genetic correlations with blood feeding.
3. Adult female longevity is greater in the selected line than in the control line, both in the presence and in the absence of a host.

## 2 Materials and Methods

### 2.1 Biting Propensity and Selection

A previous paper describes the establishment and propagation of the selected and control lines in detail (Bradshaw et al. 2022). Briefly, biting propensity was assayed after at least two generations of lab‐rearing to mitigate field effects. Through all generations of selection, including the control line that was not offered a host, hatch were placed on short days (*L*:*D* = 10:14) at 21°C to synchronize each generation in diapause in order to mitigate inadvertent direct selection on development time, generation time, or the timing of reproductive allocation. Populations were maintained as diapausing larvae on an 8:16 light:dark cycle at 21°C. To terminate diapause and sustain development, larvae, pupae, and resulting adults were exposed to long days (*L*:*D* = 18:6) and thermal conditions mimicking mid‐summer conditions in pitcher‐plant leaves at Wilma (Bradshaw et al. 2004, Figure 2A,B): a sinewave thermoperiod with a maximum temperature of 35°C and a minimum temperature of 15°C, which lagged the light cycle by 3 h. Larvae were reared in distilled water at a density of 35 larvae in 25 × 150mm Petri dishes, and were fed ad lib. A 4:1 mixture by volume of ground freeze‐dried brine shrimp (San Franciso Bay Brand) and guinea pig chow (Geisler Guinea Pig Chow, Sergeant's Pet Care Products) once a week. Adults were offered a continuous supply of organic (pesticide free) raisins as a carbohydrate source. To determine the incidence of biting, ≥ 390 individuals were reared to adults as above. Starting at first eclosion, adults were offered an anesthetized rat for 15 min three times per week between 1200 and 1400 subjective time (25°C–30°C) to minimize any circadian variation in propensity to bite (specific IACUC Protocols given below). Any female with an engorged abdomen was scored as a biter, removed from the cage, counted, and discarded. When females aggregated on the host, any female with a bent labium was scored as a biting individual and was removed from the cage and discarded. The number of adult females emerging into the adult population was determined by sexing pupal exuviae. The incidence of biting was then calculated by dividing the number of biting females by the total number of eclosing females.

**FIGURE 2:**
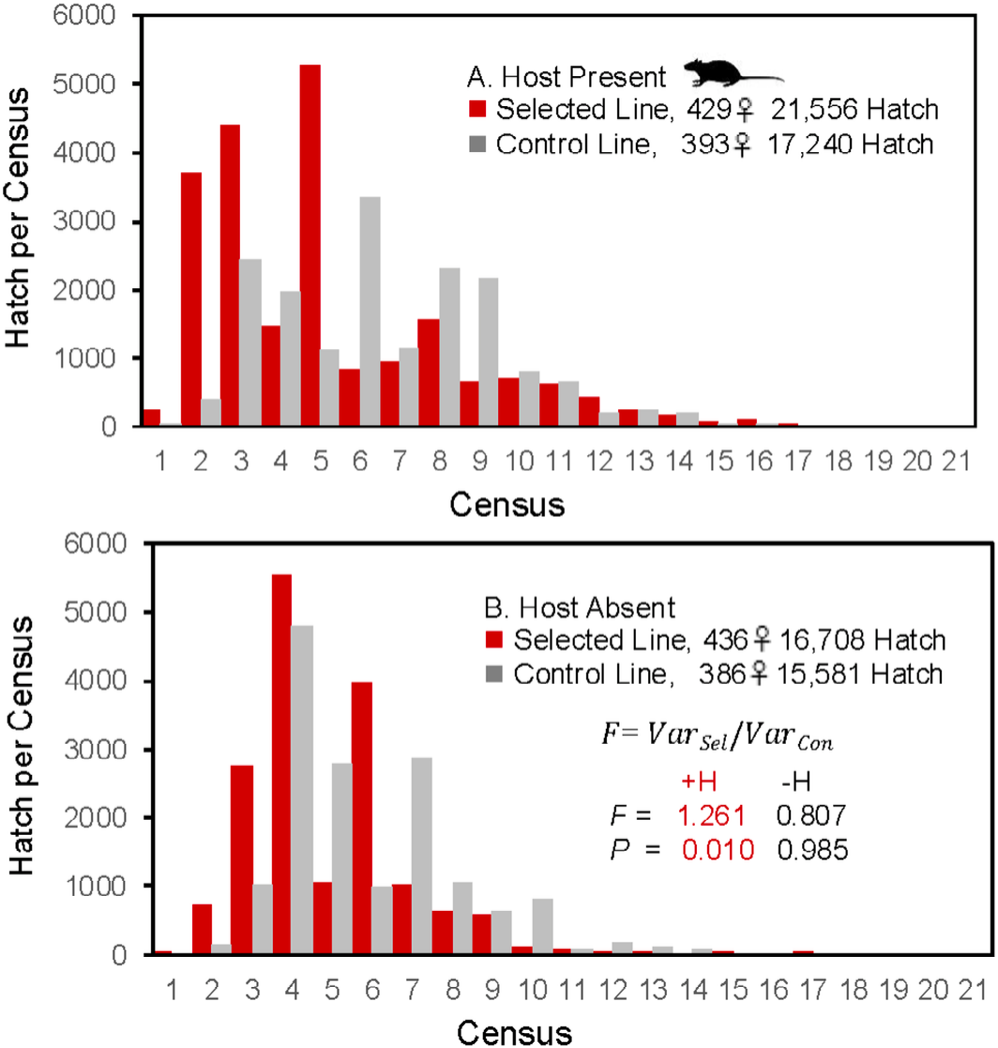
Dispersion (variance) in time of offspring hatch is greater in the selected line than in the control line in the presence (A) but not in the absence of a host (B). Inset in B: Var., variance in timing of offspring hatch in the presence (+*H*) or absence (−*H*) of a host (rat) for blood feeding. *F* and *p*, variance ratio and associated *p*‐value. Analytic details in Appendix S1.

Selection for biting began using approximately 9000 individuals from the Wilma population (Bradshaw et al. 2018, 2022), allocated to four 19 L cages, and exposed to a host (rat) three times per week between 1200 and 1400 h subjective time. The environment and protocols used for selection were as above except (1) biters were removed from their cage and placed into a separate “biting” cage with supplemental males from the same generation of the selected line, and (2) a host continued to be offered to females in the biting cage for their lifetimes. All hatch from the biting cage were used to generate the subsequent generations. Initially, hatch from biting females were not sufficient to maintain a line able to replace itself exclusively from biting individuals. In this situation, we used the abundant hatch generated by females from the same generation, but before they bit (pre‐biters), in order to augment the selected line. This protocol was followed until the selected line could sustain itself (*R*_0_ > 1.0) exclusively from biting females in the 7th generation of selection (see Bradshaw et al. 2022). Thereafter, hatch in excess of 5000 were used in experiments. The number of biting females per generation of selection ranged from 842 biting females in the F3 to 3773 in the F6, with a harmonic mean, *H* = 770 biting females per generation of selection through the F10. The control line was maintained at 1000 larvae per generation. We did not keep track of the number of males; but, with this number of females we observed no evidence of inbreeding depression in the selected or control line (Bradshaw et al. 2022). Through all generations of selection, including those that were not offered a host, hatch were placed on short days (*L*:*D* = 10:14) at 21°C to synchronize each generation in diapause. After adults of a given generation had died, their offspring were transferred to long days and reared to adulthood, as above. In a previous study, we showed that after 14 generations of selection, the biting frequency of the selected line increased to over 80%; in contrast, the biting frequency of the control line remained constant (approximately 20%) across 30 generations (Bradshaw et al. 2022).

“When hatch of a given generation in the selected or control line exceeded a desired population size, the line was thinned or experimental animals removed using a **‘**histogram’ method to minimize unintentional selection on development time in any line” (Bradshaw et al. 2022, section 2.2). Briefly, in order to thin by one third, all of the larvae in Petri dishes from one generation are lined up by date of hatch, from early to latest hatch, and every third Petri dish removed and discarded. Larvae removed for thinning or for experiments or their progeny were never returned to the selected line or to the control line.

### 2.2 Life History

Populations used for life‐history analyses were offspring of control and 11th generation selected‐line females maintained under common garden (i.e., standardized environment) conditions without access to blood. These offspring were allocated to four separate treatments: (1) the selected line exposed to a host three times per week throughout their entire adult lifetimes, (2) the control line exposed to a host three times per week throughout their entire lifetimes, (3) the selected line never exposed to a host, and (4) the control line never exposed to a host. To keep parent age and reproductive status constant and to obtain experimental first instars all hatching on the same day, eggs for each treatment were obtained from mothers never exposed to a blood source. Selected and control populations each consisted of cohorts of 980^4^ first instar larvae hatched and collected on a single day and reared in the same manner described in the above section, but with a maximum daily temperature set to 35°C, a moderate summer maximum in pitcher‐plant leaves at the Wilma locality (Bradshaw et al. 2004). Eggs were floated on distilled water in 150 × 25 mm Petri dishes. First instar hatch were reared at 35 larvae in distilled water in 150 × 25 mm Petri dishes and fed ad lib. a 4:1 mixture by volume of ground guinea‐pig chow and freeze‐dried brine shrimp. Feeding was evaluated in each individual dish three times per week. Optimal feeding generated noticeable frass pellets (starved larvae break apart the pellets contained in the delicate peritrophic membrane) without clouding the water. Clouded water or dispersed frass pellets prompted a change to a fresh dish with fresh food and fresh water. Pupae were removed to 75 mL of distilled water in open cups in 19 L adult cages. Eclosing adults were sexed by examining the terminal segment of pupal exuviae. Dead adults were sexed by the presence of male terminalia or presence of spermathecae in dissected abdomens. Eggs, first instar hatch, pupae, and pupal exuviae were collected, pupal exuviae were sexed, and counts recorded three times a week. Because of rapid decomposition, deceased adults were collected and sexed daily for the duration of the experiment. Experimental treatments involving a host for blood feeding followed the protocol as in selection, except biters were not removed from the cage.

### 2.3 Analyses

Pre‐adult development time and total lifespan were scored as days from hatch to adult eclosion and to adult death, respectively. Adult female longevity was then calculated as the average age at death minus the average age at adult eclosion. Variances were based on sums of squared individual deviations from the sample mean (Equation 1)

(1) $$\text{Variance}=\frac{\sum \left(H_{i}\right){\left(x_{i}-\bar{x}\right)}^{2}}{\sum H_{i}-1}$$

where *H*_*i*_ = number of offspring hatched to parents aged *x*_*i*_ and *x*_i_ = age of parents (days from parent to offspring hatch) in the *i*‐th census in each of the four treatments (selected or control, with or without exposure to a host). Total hatch ranged from *n* = 15,000–22,000 per treatment.

Censuses were conducted MWF or TTS throughout the adult lifetimes within each experimental treatment.

Skew and kurtosis were calculated as *g*_1_ and *g*_2_, and their standard errors estimated as √(6/*n*) and √(24/*n*), respectively, for large sample sizes > 150 (Sokal and Rohlf 1995, box 6.2, 7.1).

Standard errors of the difference between two means were estimated as the square root of the sum of their respective error variances (Sokal and Rohlf 1995, box 13.4; Welch 1947, eq. 25):

(2) $$s_{\left({\bar{Y}}_{1}-{\bar{Y}}_{2}\right)}=\sqrt{\frac{s_{1}^{2}}{n_{1}}+\frac{s_{2}^{2}}{n_{2}}}$$

where ${\bar{Y}}_{i}$ = sample mean, $s_{i}^{2}$ = sample variance, and $n_{i}$ = sample size. Tests of significance used “Welch's appoximate *t*‐test of equality of the means of two samples whose variances are assumed to be unequal” in a 1‐tailed test (Sokal and Rohlf 1995, box 13.4):

(3) $$t_{s}^{\prime }=\frac{\left({\bar{Y}}_{1}-{\bar{Y}}_{2}\right)}{s_{\left({\bar{Y}}_{1}-{\bar{Y}}_{2}\right)}}$$

Degrees of freedom for the approximate *t*‐test were calculated as in Welch (1947, eq. 29):

(4) $$f=\frac{{\left({\Sigma \lambda }_{i}s_{i}^{2}\right)}^{2}-2\left(\Sigma \frac{\lambda_{i}^{2}s_{i}^{4}}{f_{i}+2}\right)}{\Sigma \frac{\lambda_{i}^{2}s_{i}^{4}}{f_{i}+2}}$$

where *λ*_*i*_ = 1/*n*_*i*_, $f_{i}$= (*n*_*i*_ *−1*) and $s_{i}^{2}$ = sample variance. Conservatively, the minimum number of females sampled for total lifespan was used for *n*_*i*_ (*n*_*i*_ = 363–390).

Genetic variation in dispersal of offspring was evaluated separately in the presence or absence of a host from the variance ratio:

(5) $$F=\frac{{\text{Variance}}_{\text{Selected Line}}}{{\text{Variance}}_{\text{Control Line}}}$$

Degrees of freedom were calculated as (*n*_*i*_—1), where *n*_*i*_ = number of females having eclosed as adults in the *i*‐th comparison (Sokal and Rohlf 1995, sec. 8.3). The means and variances were based on the timing of individual offspring hatch (*n*_*i*_ = 15,000–22,000); significance testing was evaluated more conservatively, by using the number of eclosed females (*n*_*i*_ = 386–436) for treatment sample size (*n*_*i*_). A significant *F‐*value in Equation (5) indicated greater iteroparity in the selected than in the control line either in the presence or in the absence of a host.

Skew and kurtosis were calculated as *g*_*1*_ and *g*_*2*_ and their standard errors estimated as √(6/*n*) and √(24/*n*), respectively, for large sample sizes > 150 (Sokal and Rohlf 1995, box 6.2, 7.1).

All calculations were performed using Excel in Office 15 with the Data Analysis Toolpak.

### 2.4 Animal Use

Rats were housed in the university animal care facility (Office of Veterinary Services and Animal Care, OVSAC), handled, anesthetized, used to feed mosquitoes, and returned to OVSAC after recovery from anesthesia by students trained and certified by OVSAC for animal care and for use of controlled substances for the 11 generations of selection, according to IACUC Protocols 10‐11 and 13‐15, each with a one‐year extension; protocols were initially approved 1 August 2010 and expired 31 July 2016.

## 3 Results

Differences in variance, skew, kurtosis, or female longevity between the line selected for blood feeding and the unselected control line represent genetically correlated responses to direct selection on blood feeding (Box 1).

### 3.1 Reproductive Allocation

#### 3.1.1 Hypothesis at Risk 1

Variance: In the presence of a host, the variance in the timing of offspring production was greater in the selected than in the control line (Figure 2A: +*H*, *F* > 1.00; Appendix S1, Table S1A). In the absence of a host, there was no significant difference in the variance of offspring production, and the nonsignificant trend was towards an increase in variance in the control relative to the selected line (Figure 2B: −*H*, *F* < 1.00; Appendix S1, Table S1A).

#### 3.1.2 Hypothesis at Risk 2

**FIGURE 3:**
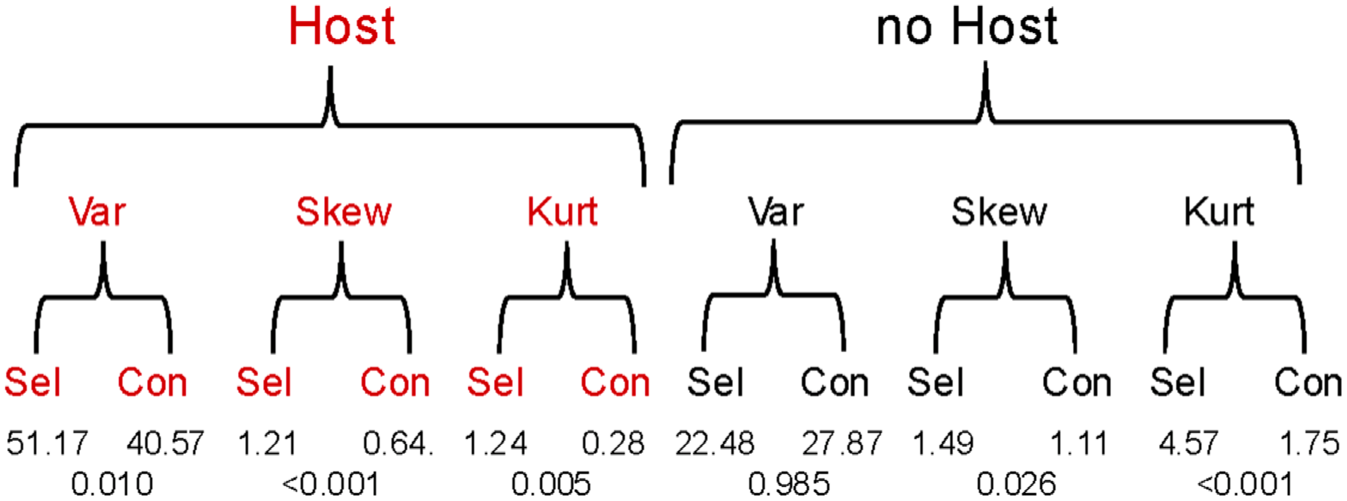
Direct selection on blood feeding results in genetically correlated responses of variance, skew, and kurtosis in the timing of offspring production. All 12 individual skew and kurtosis are individually and significantly positive. Bottom row: *p*‐values from *F*‐tests for variance ratio or *t*‐tests for skew and kurtosis between the selected and the control line. Analytic details in Appendix S1. Con, control line; Kurt, kurtosis; Sel, selected line.

In both the presence and absence of a host, skew and kurtosis were greater in the selected than in the control line (Figure 3; Appendix S1, Table S1B). Positive skew results from a tail to the right; positive kurtosis results from a symmetrical, repulsed, or spread distribution (Sokal and Rohlf 1995, 114).

#### 3.1.3 Hypothesis at Risk 3

**FIGURE 4:**
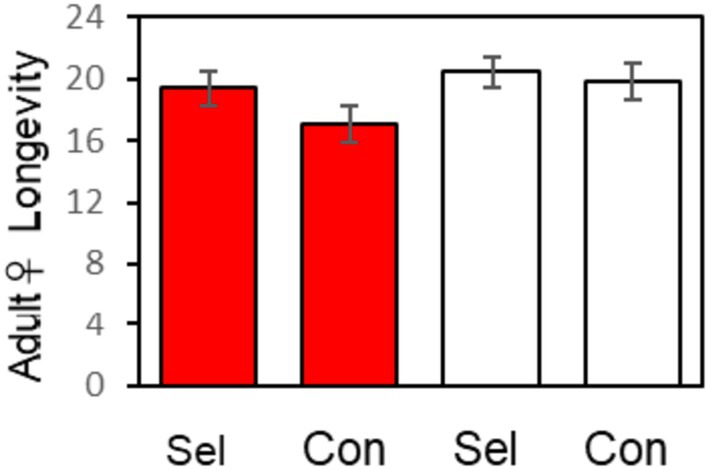
Adult female longevity is marginally greater in the selected than in the control line when a host is present (red) than when a host is absent (white). The plot shows differences between the line selected for biting and the unselected control after 11 generations of selection. Analytic details in Appendix S2.

Female longevity: Relative to the unselected control line, selection on blood feeding results in greater adult female longevity in the presence, but not in the absence of a host (Figure 4; Appendix S2, Table S2A,B): Host present, selected–control longevity = 2.30 days (*t*_*s*_ *'* = 2.84, df = 765, *p* = 0.002); host absent, selected–control longevity = 0.56 days (*t*_*s*_ *'* = 0.725, df = 726, *p* = 0.234). This 2.30 days increase in adult female longevity in the selected line relative to the control line in the presence of a host is statistically significant, but subject to type II error due to a small effect size.

## 4 Discussion

### 4.1 To Bite or Not to Bite

A low propensity to bite among mosquitoes has been correlated with relatively nutrient‐rich and predator‐free larval habitats and with a limited availability of vertebrate hosts for adults (Corbet 1964, 1967; Lounibos et al. 1982; O'Meara 1985a; Clements 1992, Ch. 23; Bradshaw 1986). Even aggressively biting, invasive species such as *Aedes aegypti* and *A. albopictus* include females that may produce some fertile eggs without a blood meal under conditions of low larval density and high larval nutrition (Mori et al. 2008; Ariani et al. 2015; Aardema and Zimmerman 2021). However, in all cases, whether it be aggressive or low‐level biting, there are both extrinsic and intrinsic costs to blood feeding due to finding and consuming a blood meal. Extrinsic costs include locating a vertebrate host and surviving consumption of a blood meal (Edman and Scott 1987; Darbro and Harrington 2007; de Silva et al. 2014). Intrinsic costs include (1) altering metabolic pathways in advance of a blood meal, whether or not blood is actually consumed (Bradshaw et al. 2018; Siperstein et al. 2022; Marzec et al. 2023), (2) dealing with heat stress incurred by imbibing hot vertebrate blood (Benoit et al. 2011; Lahondère and Lazzari 2012), and (3) metabolizing hemoglobin, especially heme, that imposes potentially lethal oxidative stress (Pascoa et al. 2002; Esquivel et al. 2014; Graça‐Souze et al. 2006; Nikbakhtzadeh et al. 2016). Indeed, when selection on biting in *W. smithii* is relaxed, the high incidence of biting (Figure 1) reverts rapidly to the original, pre‐selection level (Bradshaw et al. 2022). Thus, blood feeding does not constitute a “free lunch.” However, after a hiatus in selection for biting in the 19th generation and then renewed selection for biting in the 27th to 30th generations, we found a strong response to renewed selection in the biting line, but no change in propensity to bite in the control line (Bradshaw et al. 2022). Thus, biting propensity is a genetically variable trait that responds to repeated direct selection and is genetically correlated to life‐history traits affecting fitness.

If selection is imposed on a given trait and there is a correlated response in a second, unselected trait, then three things must be true: both the selected and unselected traits have non‐zero heritabilities and there is a non‐zero genetic correlation between them (Box 1). Consequently, among females, there is a positive genetic correlation between blood feeding and adult longevity in the presence of a host only (Figure 4). Blood‐feeding genotypes live marginally longer as adults than non‐biting genotypes in the presence, but not absence, of a host. Since the presence or absence of a host is an environmental effect, there are both genetic and genetic‐dependent environmental effects of blood feeding on adult longevity, representing genotype‐by‐environment interaction.

It is important to recall that we are focusing on variance in the timing of offspring production as the correlated trait of interest in our analyses. Furthermore, both positive skew and positive kurtosis function to increase variance relative to a normal distribution. Hence, the increase in variance in the selected relative to the control line is due to genetic variation (heritability) of variance itself, in concert with Bonner's fundamental concept of range variation (Bonner 1965, 95), and due, at least in part, to a positive genetic correlation of skew and kurtosis with blood feeding (Figure 3).

### 4.2 Genetic Variation From Among to Within Populations

Among northern and high‐altitude populations of *W. smithii* , adult females live shorter lives and are obligate non‐biting; among southern populations, adult females live longer and exhibit a low to moderate frequency of blood feeding (Bradshaw and Holzapfel 1983a; Bradshaw 1986; Bradshaw et al. 2022). Within the southern Wilma population, avid blood‐feeding genotypes live marginally longer as adults and realize a greater dispersion in offspring production than reluctant blood‐feeding genotypes (Figures 2, 3, 4). Hence, the genetic basis for the northward decrease in longevity and loss of blood feeding among populations is represented by an extension of genetic variation and covariation within a single southern population. Evolution has proceeded “along the genetic lines of least resistance” (Schluter 1996, 310).

### 4.3 Iteroparity and Spreading of Risk

In essence, iteroparity represents an observed pattern of reproductive allocation, with bet‐hedging or spreading of risk being the inferred selective process. Den Boer (1968, 178) posed the hypothesis that “density fluctuations in natural populations are stabilized to some degree by spreading of risk.” Consistent with Den Boer's hypothesis, as the summer progresses in nature at Wilma, fluctuation in population biomass of *W. smithii* decreases, as does the variation in biomass among individual host leaves (Bradshaw and Holzapfel 1983a). Reproductive allocation (Figure 2), as measured by variance in timing of offspring production (hatch), was dispersed more between the selected and control line in the presence, but not in the absence of a host (Figure 2B, inset). “Spreading the risk” of reproduction within the Wilma population involves genetic effects (selected vs. control line), environmental effects (host present vs. absent), and genotype‐by‐environment interaction (selection × host interaction). The genetic variation and covariation within the Wilma population, placed within its ecological context, reaffirms the earlier conclusion that the “functional role of facultative iteroparity thus provides a means for females developing under predictably impoverished but irregularly opportunistic conditions to [physiologically] reallocate and temporally diversify their reproductive effort” (Bradshaw 1986, 477), that is, hedge their bets.

Previous studies have considered the ecological significance of “spreading of risk” in the context of skip oviposition behavior of *W. smithii* (Mogi and Mokry 1980) and container‐breeding mosquitoes more generally (Reinbold‐Wasson and Reiskind 2021). The novelty of our current results is in establishing that this behavior is genetically correlated with blood feeding. We propose that blood feeding, usually associated with increased lifetime fecundity in vector species (Corbet 1967; Spielman 1971; O'Meara 1985a; Briegel and Hörler 1993), simultaneously reaps an under‐appreciated, reinforcing benefit of spreading the risk of reproductive failure. *W. smithii* provides an example of, at best, a meager correlated increase in fecundity to direct selection on blood feeding (Figure 4), as contrasted with a clear and significant increase in the variance in timing of offspring (Figure 2). Hence the two processes are separable, and readily recognizable as such in *W. smithii* , underscoring genetic variation in spreading of risk and as a genetic correlate of blood feeding.

Selection on lifetime fecundity would not necessarily negate spreading of risk, but rather be positively associated with repeated, blood‐fueled ovarian cycles. Reinforcement of selection on spreading the risk of reproductive failure would be especially important in populations whose larvae encounter high levels of not only density‐dependent constraints as in *W. smithii* , but also predation (Den Boer 1968, sec. III.5; Bradshaw and Holzapfel 1983b; Wilbur 1984), interspecific encounter (Lounibos 1981), desiccation (Pittendrigh 1950; McLachlan and Cantrell 1980; Bradshaw and Holzapfel 1988; Khatchikian et al. 2010), freezing (Bradshaw and Holzapfel 1991; Bergland et al. 2005), parasitism (Hawley 1985), wind exposure (Heard 1994) or the variety of environmental hazards leading to egg‐ or seed‐banking (Evans and Dennehy 2005).

## 5 Conclusions

Direct selection for blood feeding caused a genetically correlated response, not so much in increased fecundity or an extended adult female lifetime, but rather in a dispersed reproductive allocation, spreading the risk of reproductive failure. Hence, although blood feeding is usually interpreted as a mechanism that increases lifetime fecundity, our results from *W. smithii* show that blood feeding independently increases iteroparity and the potential to mitigate the risk of reproductive failure. We propose that “spreading of risk” has been and/or continues to reinforce selection on blood feeding, even in species or populations wherein blood feeding is necessary for reproduction or simply enhances lifetime fecundity. We conclude that blood‐feeding, marginal adult longevity, and dispersed reproductive allocation form a genetically coordinated life‐history syndrome that should be investigated as a key target of selection in other biting insects, especially those experiencing uncertain larval environments. Importantly, this combination of traits should enhance vectorial capacity and make more difficult vector control through larval source reduction.

## Author Contributions

**Rudyard J. Borowczak:** conceptualization (equal), investigation (equal), methodology (equal). **Mary A. Wood:** methodology (equal), writing – original draft (equal), writing – review and editing (equal). **William E. Bradshaw:** conceptualization (equal), formal analysis (equal), project administration (equal), resources (equal), writing – original draft (equal), writing – review and editing (equal). **Peter A. Armbruster:** funding acquisition (equal), writing – original draft (equal), writing – review and editing (equal). **Christina M. Holzapfel:** conceptualization (equal), funding acquisition (equal), project administration (equal), supervision (equal), writing – original draft (equal), writing – review and editing (equal).

## Conflicts of Interest

The authors declare no conflicts of interest.

## Supporting information


Appendix S1.

Appendix S2.

Appendix S3.

## Data Availability

Raw data are available as Appendices S1–, S3 uploaded as [Link], [Link], [Link] for review and publication.

## Endnotes

^1^
Latin: *itero*, spread out; *semel*, once, a single time; *paro*, bring forth, give birth to (Wikipedia).

^2^
We attribute this quote originally to Charles Elton, but we cannot find a specific reference to it. The most original usage we can cite is in McLachlan and Cantrell (1980).

^3^
Unless otherwise defined, we measured fecundity as the number of first instar hatch per eclosed female.

^4^
Petri dishes were maintained inside standard, open‐sided, stackable crates that held four stacks of nine dishes, each with 35 larvae per dish, totaling 980 larvae per crate.
