# Supplementary material for: Enhanced Iteroparity Is a Correlated Response to Direct Selection on Blood Feeding in a Mosquito
Source: Ecol Evol. 2025 Apr 28;15(4):e71335. doi: 10.1002/ece3.71335 (PMC12037228; doi:10.1002/ece3.71335)
Supplement: Supplementary file 1 — Appendix S1. [file ECE3-15-e71335-s003.docx]

Appendix I. Variance, skew, and kurtosis in the distribution of hatch (Figures 2-3): Correlated response to direct selection on blood feeding. The tables show differences between the selected and the control line (genetic differences) in variance, skew, or kurtosis nested within opportunity to consume blood (±Host).

| Table IA. Variance. | | | | |
| --- | --- | --- | --- | --- |
| Criterion | Variance | | | |
| ±Host | Host | | no Host | |
| Line | Sel | Con | Sel | Con |
| Variance | 51.17 | 40.57 | 22.48 | 27.87 |
| ♀♀ | 429 | 393 | 436 | 386 |
| *F* | 1.26 | | 0.81 | |
| *P* | 0.0097 | | 0.9852 | |
|  |  |  |  |  |

| Table IB. Skew and kurtosis. | | | | | | | | |
| --- | --- | --- | --- | --- | --- | --- | --- | --- |
| ±Host | Host | | | | no Host | | | |
| Criterion | Skew | | Kurtosis | | Skew | | Kurtosis | |
| Line | Sel | Con | Sel | Con | Sel | Con | Sel | Con |
| Value | 1.21 | 0.64 | 1.24 | 0.277 | 1.494 | 1.112 | 4.571 | 1.746 |
| **♀♀** | 429 | 393 | 429 | 393 | 436 | 386 | 436 | 386 |
| SE | 0.1183 | 0.1236 | 0.2365 | 0.2471 | 0.1173 | 0.1247 | 0.2346 | 0.2494 |
| *T* | 10.23 | 5.18 | 5.24 | 1.12 | 12.74 | 8.92 | 19.48 | 7.00 |
| *P* | <0.001 | <0.001 | <0.001 | <0.001 | <0.001 | <0.001 | <0.001 | <0.001 |
| Sel - Con | 0.57 | | 0.963 | | 0.382 | | 2.825 | |
| SE Dif | 0.1710 | | 0.3421 | | 0.1712 | | 0.3424 | |
| *T* | 3.333 | | 2.815 | | 2.231 | | 8.251 | |
| *P* | 0.0009 | | 0.0050 | | 0.0259 | | 6.23E-16 | |
