# Supplementary material for: Enhanced Iteroparity Is a Correlated Response to Direct Selection on Blood Feeding in a Mosquito
Source: Ecol Evol. 2025 Apr 28;15(4):e71335. doi: 10.1002/ece3.71335 (PMC12037228; doi:10.1002/ece3.71335)
Supplement: Supplementary file 2 — Appendix S2. [file ECE3-15-e71335-s001.docx]

Appendix II. Adult female longevity (A) calculated as the difference between total lifespan and preadult development time and (B) evaluated using Welch’s approximate t-test (cf. Figure 4).

|  |  | | | | |  |
| --- | --- | --- | --- | --- | --- | --- |
|  | Table IIA. Summary input data. | | | |  |  |
|  |  | Sel + Host* | Con+Host | Sel no Host | Con no Host |  |
|  | Female Lifespan from hatch to death | | |  |  |  |
|  | N** | 384 | 383 | 390 | 363 |  |
|  | Mean | 57.83 | 60.13 | 57.53 | 59.782 |  |
|  | Variance*** | 103.57 | 99.531 | 86.131 | 101.767 |  |
|  | St. Error | 0.519 | 0.51 | 0.47 | 0.529 |  |
|  | Female pre-adult Development Time from hatch to adult eclosion | | | | |  |
|  | N | 429 | 393 | 436 | 386 |  |
|  | Mean | 38.45 | 43.05 | 37.09 | 39.9 |  |
|  | Variance | 17.982 | 30.551 | 11.417 | 23.761 |  |
|  | St. Error | 0.205 | 0.278 | 0.162 | 0.248 |  |
|  | Adult female Longevity = lifespan - pre-adult Development Time | | | | |  |
|  | N | 813 | 776 | 826 | 749 |  |
|  | Mean | 19.38 | 17.08 | 20.44 | 19.882 |  |
|  | Variance | 121.552 | 130.082 | 97.548 | 125.528 |  |
|  | St. Error | 0.563 | 0.583 | 0.500 | 0.588 |  |
|  |  |  |  |  |  |  |
| *Sel, selected line; Con, Unselected control line | | | | | | |
|  | | **N = N_Lifespan_ + N_Development Time_  ***Variance_Adult Lonngevity_ = Var_Lifespan_ + Var_Deveopment Time_ | | | |  |

Assumes no Cov (Y_1_, Y_2_), (Sokal & Rohlf, 1995, Eq. 15.9a)

| Table IIB. Calculating the difference in adult female longevity between the selected (Sel) | | | | | |
| --- | --- | --- | --- | --- | --- |
| and the control (Con) line in the presence (red) or absence (black) of a host. | | | | | |
| Variable | Sel + Host | Con + Host | Sel no Host | | Con no Host |
| $\bar{Y}_{i}$ | 19.38 | 17.08 | 20.44 | | 19.88 |
| Min *n_i_*  (♀♀) | 384 | 383 | 390 | | 363 |
| *λ_i_=*1*/n_i_* | 0.00260 | 0.00261 | 0.00256 | | 0.00275 |
| s_i_^2^ | 121.55 | 130.08 | 97.55 | | 125.53 |
| *f_i_ = n_i_* - 1 | 383 | 382 | 389 | | 362 |
| A | 0.4306 |  | 0.3551 | |  |
| B | 0.000561 |  | 0.000489 | |  |
| *f* ** | 765.98 |  | 724.94 | |  |
| *df* | 765 |  | 724 | |  |
| Sel - Con* | 2.30 | *±0.810* | 0.56 | | ±*0.722* |
| *t*_s_’ | 2.839 |  | 0.725 | |  |
| *p* (1-tail) | 0.0023 |  | 0.2342 | |  |
|  |  |  |  |  |  |
| *** Difference *± Standard Error of the Difference* | |  |  |  |  |
| **Proofing calculation of *f* in Excel is simplified if the equation is broken down | | | | | |
| into A and B elements so that *f* = (A-2B)/B | | |  | |  |
|  |  |  |  |  |  |
| Calculations from: Sokal and Rohlf 1995, Box 13.4; Welch 1947 | | |  | |  |
|  | |  |  | |  |
